# Why did some viruses evolve to be giants while others did not?

**DOI:** 10.1073/pnas.2610442123

**Published:** 2026-09-11

**Authors:** Eugene V. Koonin, Mart Krupovic

**Affiliations:** ^a^Computational Biology Branch, Division of Intramural Research, National Library of Medicine, National Institutes of Health, Bethesda, MD 20894; ^b^ Institut Pasteur, Université Paris Cité, Cell Biology and Virology of Archaea Unit, Paris 75015, France

**Keywords:** virosphere, evolution of viruses, giant viruses, replicators, reproducers

## Abstract

Viruses are ubiquitous mobile genetic elements that are obligate symbionts of cellular life forms and replicate exclusively within host cells. The current taxonomy of viruses includes 10 realms (the top rank taxon), 8 of which have small genomes in the range of one to about 60 kilobases and commensurately small virions. However, the two realms of viruses with double-stranded DNA genomes, *Varidnaviria* and *Duplodnaviria*, include several groups of viruses known as giant viruses, with genomes reaching nearly 5 megabase pairs, larger than the genomes of the majority of bacteria and archaea, and large virions also exceeding in size many prokaryotic cells. The discovery of giant viruses has been widely claimed to blur the differences between viruses and cells, especially, because some of these viruses encode signature cellular proteins, such as many translation system components. We summarize evidence that, despite the large sizes of their particles and genomes, and vast, diverse gene repertoires, giant viruses are typical viruses that evolved from smaller ones via accretion of genes recruited from the hosts. The divide between viruses and cellular life forms lies between replicators and reproducers which is, arguably, the most fundamental gulf in biology that appears to have never been crossed. A key question on evolution of viruses is what factors drive some groups of viruses to follow the *K* evolutionary strategy, accruing numerous genes involved in virus–host interactions and reaching giant genome sizes, whereas others follow the *r* strategy, retaining compact genomes under selection for maximum replication rate.

All cellular organisms in the biosphere (with the possible exception of some highly degraded endosymbiotic bacteria) are infected by multiple viruses (1–5). Viruses are a distinct type of mobile genetic elements (MGE) that encode at least one protein that is a major component of virions (virus particles) encasing the genome of the virus (6). Viruses are obligate intracellular symbionts that strictly depend for their replication on functional systems of the host cell, in particular, the molecular machineries involved in energy transformation and protein synthesis. Viruses possess genomes that encode structural proteins of the virions as well as proteins involved in viral genome replication and, in some cases, expression, as well as proteins involved in various forms of virus–host interaction (7). In a sharp contrast with cellular organisms, genomes of viruses—that is, the genetic material incorporated into virions—can be represented by all types of nucleic acids including single-stranded (ss) or double-stranded (ds), linear or circular RNA or DNA molecules (Fig. 1) (8, 9).

**Fig. 1.**
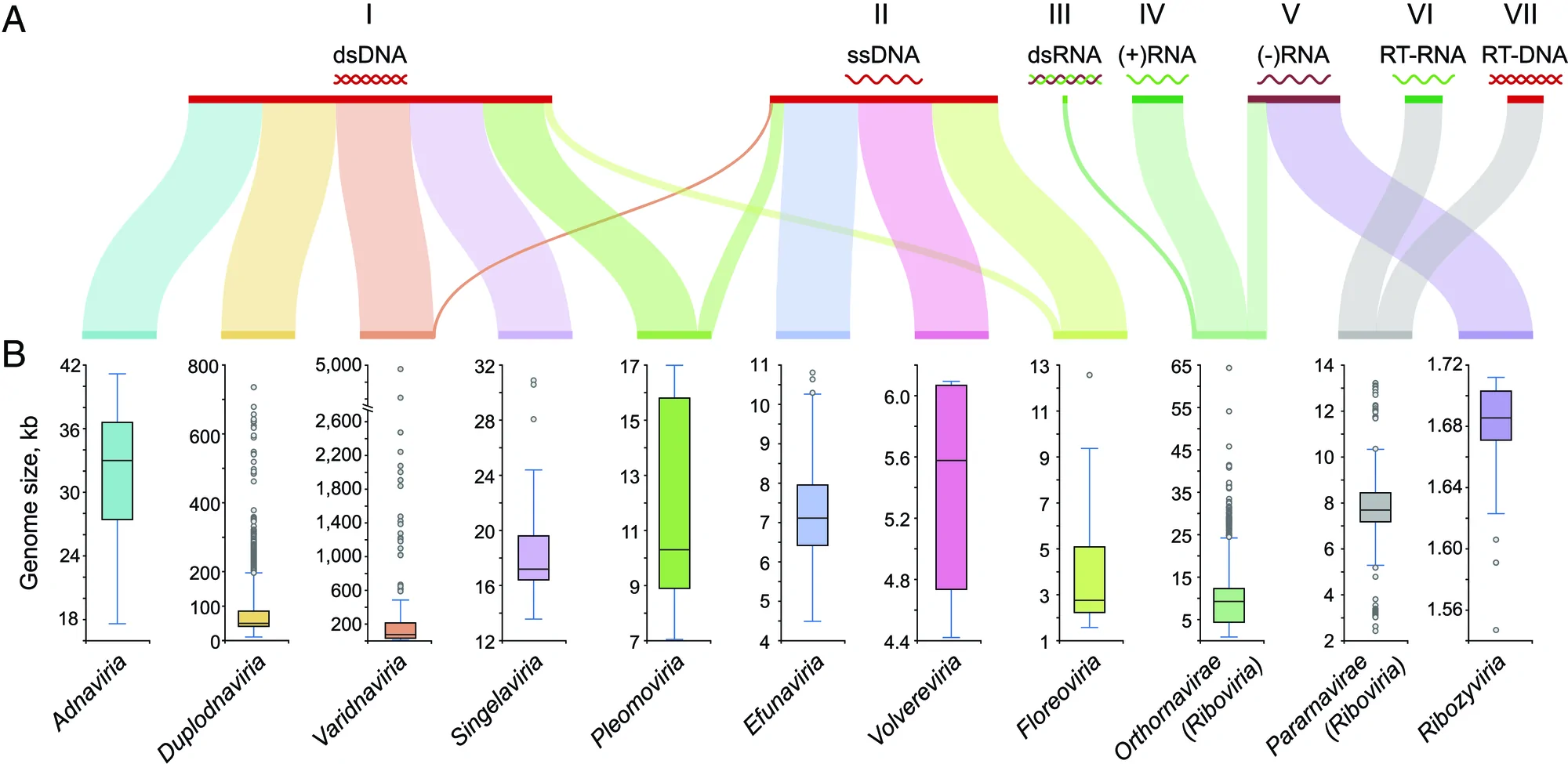
The realms of viruses and their genome size ranges. (*A*) Baltimore classes, which are defined based on the type of nucleic acid encapsidated in the virion and accordingly determining viral replication-expression cycle (8, 9), are connected to the virus realms. (*B*) Range of genome sizes (kb) is shown for each viral realm. The genome size data for classified representatives of each realm were retrieved from the NCBI GenBank database (10).

In the last decade, mining of the rapidly increasing, in size and quality, metagenomes and metatranscriptomes led to a dramatic expansion of the known diversity of viruses, and concomitant, concerted efforts in phylogenomics provided for vastly improved understanding of the evolutionary relationships among viruses and development of a comprehensive taxonomy of viruses (4). Viruses as a whole are polyphyletic, with not a single gene conserved across the entire virosphere. However at the top level of the taxonomy, viruses are classified into 10 realms, each held together by one or more hallmark genes each inferred to originate from a common ancestor (11).

Traditionally, viruses are perceived as minuscule biological entities. Indeed, the very discovery of viruses—specifically, tobacco mosaic virus—was based on the identification of a plant pathogen that passed through bacterial filters, and for decades, fractionation by size served as the basis of separation of viruses from cellular life forms (12). This paradigm holds for a broad variety of viruses. Indeed, of the 10 viral realms, 8 include viruses with small genomes (mostly RNA or ssDNA), in the range from about 2 to about 60 kilobase pairs (Kbp) (Fig. 1). This is several times smaller than even the tiniest genomes of intracellular symbiotic bacteria and more than an order of magnitude smaller than genomes of typical, free-living prokaryotes (13). However, the two realms of viruses with dsDNA genomes, *Varidnaviria* and *Duplodnaviria*, span much broader ranges of genome size, in the case of *Varidnaviria*, more than 3 orders of magnitude (Fig. 1).

Indeed, the size barrier between viruses and cellular life forms was shattered with the discovery of the giant mimivirus in 2003–2004 (14–16). With a 1.2 megabase pairs (Mbp) genome and 700 nm icosahedral capsid, mimivirus barged deep into the cellular territory, surpassing, in both the particle and genome size, numerous parasitic bacteria and archaea, and some free-living ones. In the subsequent two decades, a broad diversity of giant viruses was discovered in several branches of what is now phylum *Nucleocytoviricota* in the realm *Varidnaviria* (Fig. 2) (17–28). For a decade, pandoraviruses with genomes of about 2.5 Mbp have been holding the viral genome size record (20), but recently, with the discovery of megagenomoviruses, that record might be shattered, now reaching nearly 5 Mbp (29), larger than the majority of free-living bacteria and archaea (with the caveat that megagenomovirus genomes have been assembled from metagenomic contigs and as such should be considered provisional). With the proliferation of viral genomes across the broad size range, giant viruses often have been arbitrarily defined as those with genomes in excess of 0.5 Mbp (historically, the term “giant viruses” was coined before the discovery of the mimivirus and originally applied to algal viruses with the genomes of “merely” ~330 Kbp (30), underscoring the lack of any objective criterion of gigantism). Under this definition, numerous giant viruses, such as jumbo or mega phages (31–33) and mirusviruses (34, 35), are found also in realm *Duplodnaviria*, even though the genome sizes in excess of a megabase so far remain limited to *Nucleocytoviricota* within *Varidnaviria*.

**Fig. 2.**
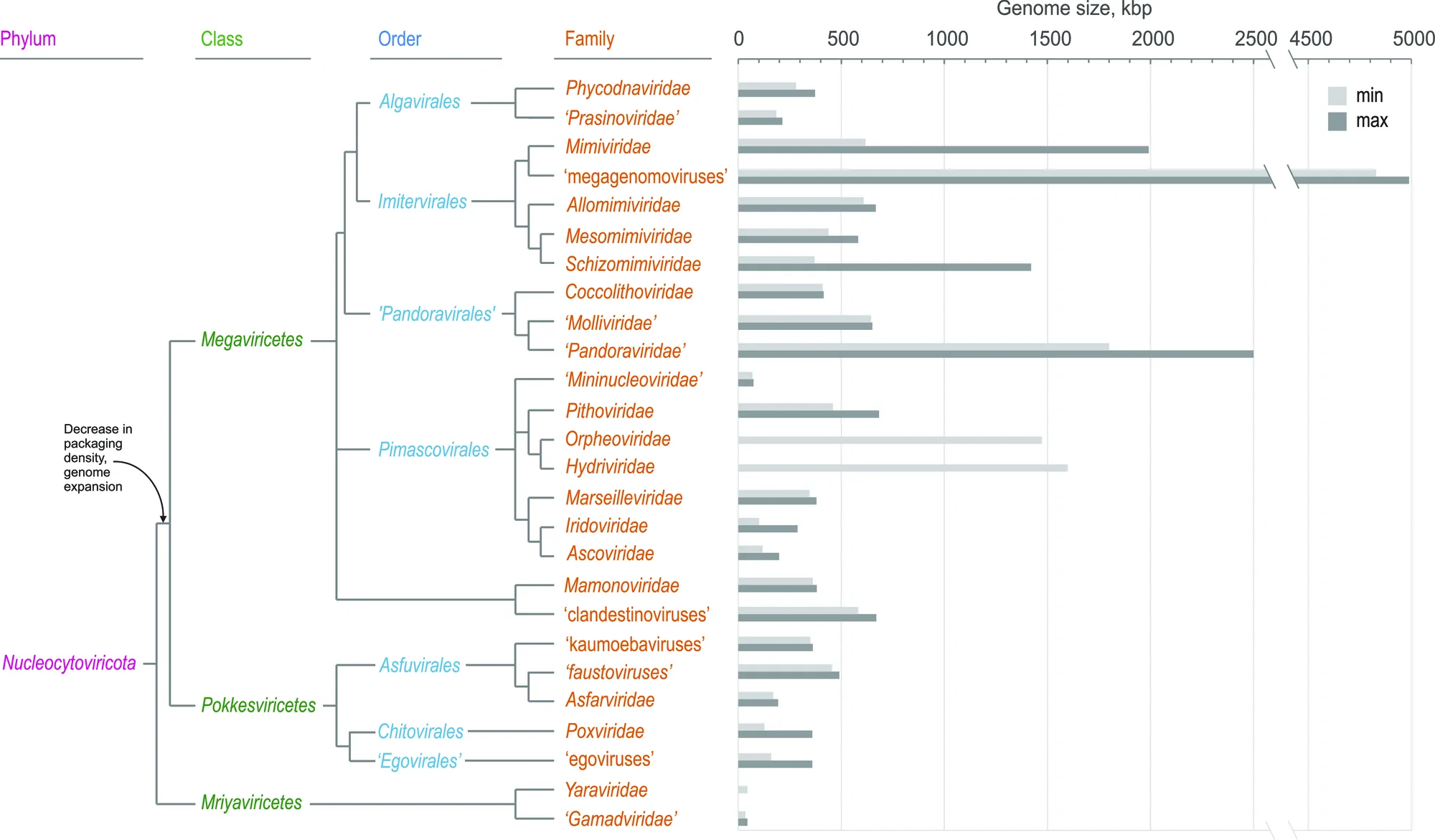
Independent evolution of giant viruses in multiple lineages of phylum *Nucleocytoviricota*. The schematic phylogeny of *Nucleocytoviricota* is based on recent analyses (25, 29, 36–38). On the right, bar plot shows the minimal and maximal genome sizes for each virus family. The data were retrieved from ref. 25 and relevant publications.

## The Nature and Origins of Giant Viruses

The discovery of giant viruses and the finding that some of them, including the mimivirus, encode multiple components of the translation system, the quintessential cellular molecular machinery, prompted recurrent statements on giant viruses blurring the boundary between cells and viruses (15, 29, 39–44). Hence, the idea that giant viruses represent a distinct type of biological entities fundamentally different from both viruses and cells, and even a special term to denote these agents has been proposed: giruses (39) (the term failed to become adopted, though). More specifically, it has been proposed that giant viruses should be considered the fourth domain of life (along with bacteria, archaea, and eukaryotes), perhaps, a degraded derivative of now extinct cells of that hypothetical domain (41, 45–47). These ideas were seemingly buttressed by the discovery of giant viruses, such as tupanvirus, that encode nearly all components of the translation system except for the ribosome (19). In a complementary development, it has been reported that the virions of the giant pandoraviruses can generate a proton gradient, seemingly breaking another barrier separating viruses from cells (48).

The ideas on the special status and potential cellular origin of giant viruses provide for fascinating discussions. Do they, however, stand the phylogenomic scrutiny? Several lines of evidence indicate that the answer should be a resounding no. First, all the peculiarities of their gene repertoires notwithstanding, giant nucleocytoviricots share with the other members of the phylum not only the hallmark genes of realm *Varidnaviria*, but a larger set of at least 20 core genes, indicative of a shared evolutionary history (24). The same applies to giant viruses in realm *Duplodnaviria*, both jumbo phages (31) and mirusviricots (35). Second, the evolution of the viral genome size can be traced through the reconstruction of the history of gene gains and losses. These reconstructions are performed by mapping the gene sets of the viruses (or cellular life forms) onto an evolutionary tree assumed to represent the history of the viruses (or organisms) as a whole and applying maximum parsiomony or maximum likelihood algorithms to infer the most probable pattern of gene gains and losses (49, 50). Such reconstructions performed for *Nucleocytoviricota* using the phylogenetic tree of concatenated sequences of proteins conserved across the phylum as the scaffold strongly suggest that giant viruses evolved on multiple occasions from ancestral viruses with substantially smaller genomes (Fig. 2) (23, 24). In general, evolution of viruses with large dsDNA genomes, particularly, nucleocytoviricots, appears to be primarily a history of gene accretion by recruitment of host genes, resulting in expansion and complexification of the genomes (although gene loss leading to reductive evolution prevails in a few lineages) (23, 24). In different lines of descent, there is a continuum of viral genome sizes from comparatively small to giant (Fig. 2). Notably, the deepest lineage of nucleocyoviricots are mriyaviricetes, with genomes of only about 40 kb, much smaller than any other members of the phylum and lacking some of the core genes (36). Even deeper ancestry can be traced back to polinton-like viruses (*Eupolintoviridae*) with genomes of about 20 kb (51, 52). Third, the signature genes of cellular life forms, such as translation system components, show scattered distribution among giant viruses (Fig. 2), and their phylogenies suggest capture of cellular ancestors from widely different hosts, at different stages of evolution (53–55). Moreover, the abundance of these genes does not generally correlate with the viral genome size: For example, pandoraviruses with giant genomes of about 2.5 Mbp have many fewer translation-related genes than mimiviruses with smaller genomes in the range of 1.1 to 1.5 Mbp (23). Furthermore, in the virosphere, translation-related genes are not exclusive to giant viruses: for example, many tailed phages (caudoviricetes) with genomes below 100 kb encode multiple tRNAs (56), and in some cases, also ribosomal proteins (57).

Increase in the genome size necessitates the increase in internal volume and, accordingly, the size of the virion. Two major strategies are employed by giant viruses. First, icosahedral capsid size can increase through discrete increments in the triangulation (*T*) number (58–60). This is the most common strategy that is used by both nucleocytoviricots and duplodnavirians. Second, some nucleocytoviricots, such as pithoviruses and pandoraviruses, have independently abandoned the ancestral icosahedral capsids in favor of superficially similar, but apparently distinct ovoid, “amphora-shaped” particles (20). In the case of pandoraviruses, the related molliviruses, which retained the ancestral major capsid protein, form spherical virions that are the likely evolutionary intermediate on the path from typical icosahedral capsids to irregularly shaped virions (61).

Taken together, these independent lines of evidence clearly show that, with all their impressive size and functional richness, giant viruses are no distinct type of biological entities, but rather, form an evolutionary continuum with other, smaller, typical viruses (62, 63).

## Reproducers and Replicators: The Fundamental Divide between Cellular Life Forms and MGEs

Even if the provocative ideas on giant viruses representing or descending from a fourth domain of cellular life have been largely put to rest by in-depth phylogenomic analyses briefly summarized in the preceding section, the notion of giant viruses “blurring the boundary between cells and viruses” (64) or “challenging the fundamental definitions separating viruses from cellular life” (29) continue to appear in prominent publications. We argue here that the boundary is solid and safe, whereas the challenge is illusory.

There are two fundamentally distinct types of evolving biological entities: reproducers and replicators (65, 66). Reproducers can be considered analogue devices that retain physical continuity throughout their evolution. All cellular life forms have the properties of reproducers as captured in the classic formula of Rudolf Virchow: *Omnia cellula e cellula* (all cells from [other] cells). Indeed, the entire history of life can be depicted as a single tree of cells in which all cells surviving at this time represent uninterrupted lineages tracing back to the last universal cellular ancestor (LUCA) (67). Replicators, in contrast, are digital devices inasmuch as their propagation and evolution require only the information contained in the nucleotide sequence of the genome and not physical continuity. All MGEs, and viruses in particular, are replicators (the propagation of many viruses including giant ones requires not only the genomic nucleic acid but also some virion protein(s) or even macromolecular structures, such as virion cores, to enter the cell; however, such structures are not transmitted to the next generation). Cellular life forms, in contrast, are fundamentally more complex entities that represent a union of reproducers and replicators, that is, the cell and the genome, respectively. The fixation of mutualistic symbiosis between primordial reproducers (protocells) and replicators (genetic elements) could have been a pivotal point in the origin of life (68).

Without exaggeration, the split between reproducers and replicators appears to be the most fundamental divide in biology. There is no evidence that the barrier between these two types of propagating, evolving entities has ever been crossed in the more than 4 billion years of the evolution of life. That is, there is no indication that a replicator ever evolved from a reproducer or vice versa. Given the tight but not blending symbiosis between reproducers and replicators perpetuating throughout the entire course of life’s evolution, this barrier appears to be fundamentally impenetrable, a key exclusion principle of biology. In light of this principle, giant viruses, all their complexity notwithstanding, remain typical replicators. No matter what functional systems might be discovered in giant viruses—for example, a complete translation system or an electron transport chain—inasmuch as they adhere to the digital mode of propagation and evolution, the divide will not be crossed. Accordingly, there is no support for scenarios of evolution of viruses, giant or not, from cells, or vice versa, although extensive gene exchange between MGE and their hosts certainly made major contributions to the evolution of both cells and viruses (69).

## Why Giant Viruses?

As discussed above, the origin of giant viruses is not particularly enigmatic, at least, in general outline: these giants of the virosphere evolved from smaller, simpler viruses via gene accretion. What we do not understand, however, are the evolutionary factors that drive some groups of viruses, such as several lineages of nucleocytoviricots, toward evolving into giants, whereas others, such as members of the other phylum within *Varidnaviria*, *Preplasmiviricota*, remain tightly constrained in size (52). Such limitations are unsurprising for viruses with RNA and ssDNA genomes given the inherent fragility of long molecules of these types, topological constraints associated with their replication and shortage of efficient repair mechanisms. Furthermore, despite the limitations, these viruses display a trend of gene accretion as well, albeit on a much smaller scale, as observed, in particular, among “giant” nidoviruses with genome reaching more than 60 kb in size (70–72). However, why genome inflation occurred independently in multiple lineages of viruses with dsDNA genomes, whereas many others remained tightly constrained and seem to be evolving under selection for genome minimization (73), is more puzzling, calling for exploration and explanation.

A useful way to conceptualize the evolution of giant viruses in some but not other lineages of *Varidnaviria* and *Duplodnaviria* seems to be the framework of the *r* and *K* selection strategies (74–78). These terms come from the logistic equation of population dynamics well known in ecology: *dN/dt* = *rN*(1 − *N/K*) where *N* is the population size, *r* is the maximum growth rate, and *K* is the carrying capacity of the local environment, that is the maximum population size the environment can sustain. The *r-*strategists evolve under selection for the highest replication rate which is advantageous in unstable, fast-changing environments with population densities well below the carrying capacity. Under these conditions, the winners in the competition are the fastest growers, and there is little benefit in complex adaptations that are likely to become useless when the environment changes. By contrast, *K-*strategists thrive in stable environments, with population densities close to the carrying capacity, where competition is intense and complex adaptations pay off in the long term.

**Fig. 3.**
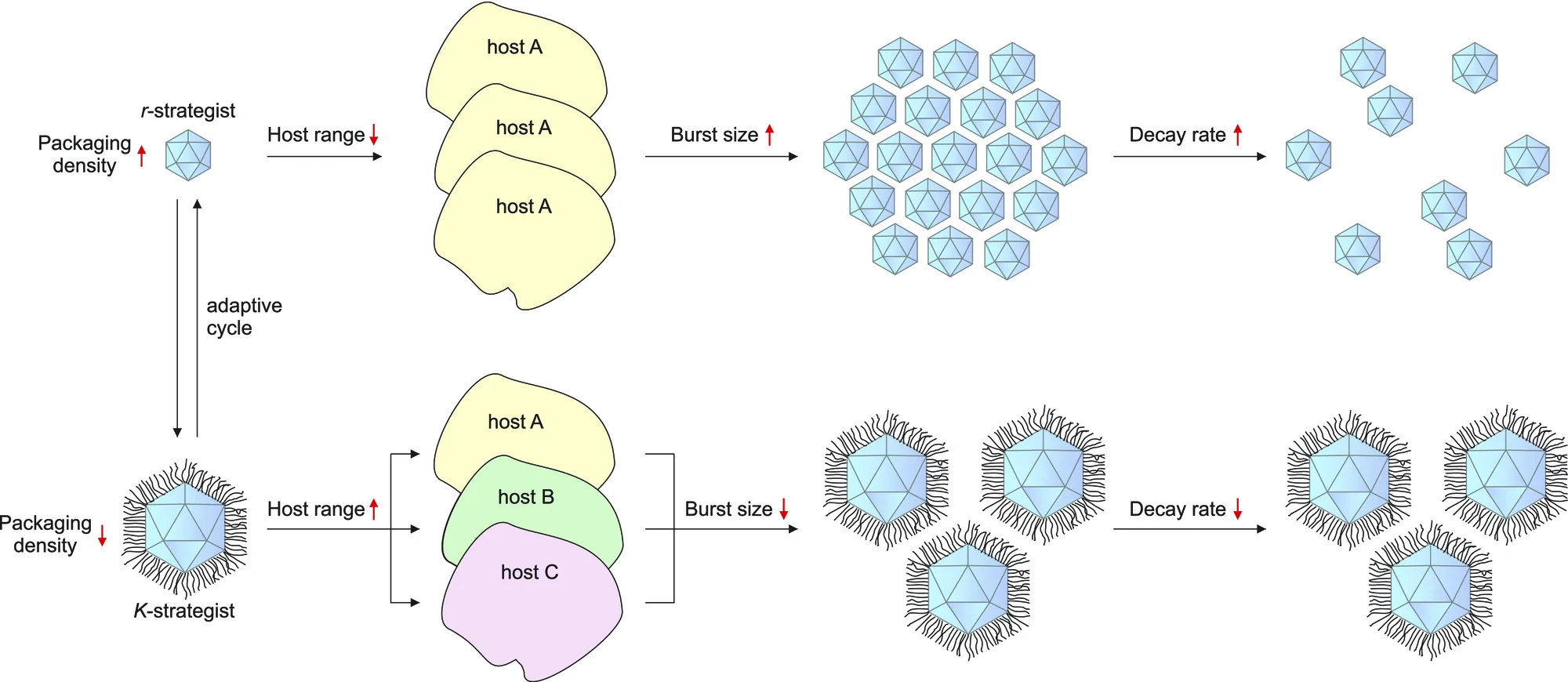
Evolution of giant viruses within the framework of the *r* and *K* selection theory. The figure depicts different traits associated with small and giant viruses (79), which evolve under *r* and *K* strategies, respectively. The reversible transition between viruses with smaller and giant genomes is denoted adaptive cycle.

There are straightforward, general advantages for viruses to be *r-*strategists. Indeed, smaller virions have higher diffusivity and accordingly greater contact rate with host cells whereas smaller genomes require less resources for replication, resulting in larger burst sizes (79). Given that actively propagating viruses can reach enormous effective population sizes, providing for strong selection (80), genome minimization associated with the *r-*strategy can be considered the default mode of virus evolution. For the switch to *K-*strategy to occur, resulting in genome expansion, the benefits of the increased number of genes must outweigh the costs. Such benefits could include increased infection efficiency, mediated by dedicated viral proteins and structures, potentially decreased decay rate and, perhaps most important, greater autonomy with respect to host cell functions, providing for expanded host range (Fig. 3). For instance, comparison of closely related prasinoviruses, algal viruses of the family *Phycodnaviridae*, revealed statistically significant, although weak, positive correlation between the host range and the size of a hypervariable region encoding various cell recognition, adhesion, and morphogenesis proteins (81). The broad host range is considered to be particularly beneficial in oligotrophic ecosystems with limited carrying capacity, which indeed are known to harbor many giant viruses (79). Many if not most giant viruses are generalists capable of infecting diverse unicellular eukaryotic hosts, arguably, as a consequence of the *K-*strategy. This broad host range is notably demonstrated by the ability of numerous nucleocytoviricots to efficiently replicate in *Acanthamoeba* or *Vermamoeba* which are not their natural hosts but have been successfully used as model systems for giant virus isolation and characterization (82–85). More specifically, at least some giant viruses enter the host cells by phagocytosis which requires large particle size in excess of about 1 µm and might have been one of the factors driving viral genome expansion (23, 86–89). Marseilleviruses, members of a nucleocytoviricot family with moderate-sized genomes, have been shown to form multivirion vesicles that, unlike individual virus particles, can be phagocytosed, apparently, an adaptation alternative to gigantism (90). Finally, viral genome expansion is not necessarily adaptive in all cases. Thus, the genomes of pandoraviruses, the largest among the isolated viruses, are infested with numerous transposons (91), suggestive of neutral genome expansion, conceptually, relating to that observed in eukaryotes.

The dichotomy between *r-* and *K-*strategies in the evolution of viruses parallels similar trends in the evolution of prokaryotes. The default regime of evolution in prokaryotes has been proposed to be genome streamlining and simplification underlain by their (typically) large effective population size (80). However, detailed analyses of the correlations between selective pressure and genome size suggest that the opposite evolutionary regime of genome expansion is even more common than streamlining in prokaryote evolution (92–94), presumably, being driven by selection for functional diversification, possibly, coupled with the evolution of generalists (95). The latest analysis of the selective landscapes of prokaryote evolution in a large genomic database revealed pronounced heterogeneity, with switches from the expansion to the streamlining regimes apparently occurring independently in multiple, even relatively narrow groups of bacteria (96). Similar detailed analysis of viral selective landscapes remains to be performed and can be expected to shed light on the driving forces of genome expansions leading to gigantism.

No particular ecological setting can fully account for the choice between the *r* and *K* strategies among viruses because both small and giant varidnavirians can coexist in the same ecological niche (e.g., aquatic environments), and some even infect the same hosts (79). It appears likely that different population states of the hosts favor different viruses, in particular, *r-*strategists vs *K-*strategists. The actual environment in which selection for particular evolutionary strategies and gene compositions of viruses takes place is the intracellular milieu rather than the extracellular space although the external environment exerts substantial indirect effects through the hosts as well as a direct effect on virions. Thus, the fascinating question of why some viruses follow the *r-*strategy and remain compact and streamlined whereas other, related ones adopt the *K-*strategy, is primarily a question on coevolution between viruses and hosts as well as between different viruses. The role of intervirus competition remains to be explored in detail, but one notable case study includes downregulation of phagocytosis in *Acanthamoeba* infected with mimiviruses which results in superinfection exclusion (89). It seems likely that active intervirus competition, a distinct form of the evolutionary arms race, occurs primarily among *K-*strategists, perhaps, driving genome expansion, but much more data are needed to test this hypothesis.

Comparative genome analysis within families of nucleocytoviricots suggested an “accordion” model under which evolution of these viruses alternates between phases of genome growth and contraction (97, 98). In the more general context of evolution of complex systems, the accordion corresponds to the adaptive cycle concept, that is, alternating *r-* and *K-*strategies constituting the basis of adaptation (99, 100) (Fig. 3). There is no specific information on the factors that tune the accordion but population dynamics of the hosts and/or changes in the viral host range likely play major roles. A question of considerable interest seems to be the potential link between *r-* vs *K-*strategy of viruses and their hosts.

The next level question is, what drove the acquisition by (giant) viruses of particular functional classes of genes, for example, those encoding translation system components. The general answer is that, to efficiently propagate in their particular hosts, these viruses benefit from supplementing and reprogramming the host translation machinery. The recent demonstration that translation initiation factor IF4F encoded by mimiviruses reprograms the translation machinery from host to viral mRNAs (64) is a striking illustration of the *K-*strategy. In general, to characterize the regimes of virus–host coevolution, a detailed investigation of both the population dynamics, the cell biology of the hosts and their response to virus are needed.

## Capsid Architecture Determines Whether Adaptation Occurs Primarily by Optimization or by Innovation

Varidnavirians infecting eukaryotes are classified into two phyla, *Preplasmiviricota* and *Nucleocytoviricota*, comprising viruses with relatively small and large genomes, respectively. Accordingly, preplasmiviricots have relatively small icosahedral capsids, whereas nucleocytoviricot virions vary in size from large to truly huge. The only known exceptions to this trend are mriyaviricetes (36), such as yaravirus (101), and mininucleovirids (102), nucleocytoviricots that have relatively small genomes and capsids, close to the range typical of preplasmaviricots. We propose that intrinsic features of virion architecture constrain the genome evolution and adaptive potential of a virus, which eventually can manifest in the choice (or lack thereof) between the *r* and *K* strategies.

Viruses with smaller icosahedral capsids typically have relatively high genome packaging densities, which is likely important for virion stability and infection (103–105). Given the limited internal capsid volume available to accommodate the genome, the high packaging density typical of small varidnavirians constrains genome expansion such that new genes (and hence functions) can be acquired only at the expense of preexisting genes. By contrast, in nucleocytoviricots with icosahedral capsids, the packaging densities are generally low, and the bigger the capsid, the lower the density (106, 107). Accordingly, new genes—and functions—can be readily added, presumably, until a certain packaging density threshold is reached. The relationship between the virion size and packaging density is likely to be even more relaxed in giant viruses with nonicosahedral virions, such as pandoraviruses and pithoviruses. Indeed, deletion of almost one-third of the pandoravirus genome (600 kb, 300 genes) did not affect virion morphology or size although viral fitness decreased (108). The lack of internal pressure in the capsids of large nucleocytoviricots enables the genome accordion dynamics (expandable genomes), whereas in small viruses, such as preplasmiviricots, only relatively small increase or decrease in genome size would be permitted (nonexpandable genomes).

The critical point in the evolution of varidnavirians was the switch from the relatively small preplasmiviricot-like capsids to the large capsids typical of nucleocytoviricots. This transition apparently happened only once, in the common ancestor of *Megaviricetes* and *Pokkesviricetes* (Fig. 2). Indeed, structural studies have shown that icosahedral capsids of viruses from both nucleocytoviricot classes are assembled on the same principles (109–112). The giant capsids are built from 20 triangular arrays (trisymmetrons) and 12 pentagonal arrays (pentasymmetrons), with the size of the triangular plate being determined by the length of the tape measure protein, which runs along the edge of neighboring trisymmetrons and fixes the distance between the fivefold vertices (111–113). This assembly mechanism clearly was inherited from a particular lineage of bacterial varidnavirians, namely, *Tectiviridae* (103, 114), the only family of prokaryotic varidnavirians included in the phylum *Preplasmiviricota*.

The bimodal distribution of capsid sizes raises the question why capsids do not simply expand commensurately with the genome size, as soon as new genes are acquired, which would result in a smooth continuum of genome and capsid sizes expanding in parallel. The answer could lie in the intrinsic complexity of icosahedral capsid assembly, which involves intricate interactions between multiple components, including MCP and penton subunits, and a complex network of minor capsid proteins required for capsid stabilization and size determination, as well as other structural components of the virion, such as a lipid membrane in most varidnavirians (115). Evolution of the capsid size (and accordingly increase in the internal capsid volume) occurs in discrete steps, characterized by changes in the *T* number, according to the quasi-equivalence theory (58–60). Conceivably, due to the high curvature of the smaller capsids, transition to a larger *T* number requires many interaction interfaces to be changed simultaneously and these changes should co-occur with the commensurate increase in the genome size to maintain optimal packaging density. Asynchronous increase of either of the two components will likely yield nonviable virions. Paradoxically, although giant capsids are vastly more complex than capsids of smaller viruses (111–113), increase in the *T* number may be less constrained, in part, at least, due to the low packaging density in giant capsids, resulting in uncoupling of the changes in the capsid size from those in the genome size. Thus, genome expansion may follow increase in the capsid volume and/or vice versa, eliminating the constraints affecting the smaller viruses. Furthermore, due to the large size of the capsids, the triangular plates forming the capsid shell are nearly planar so that addition of subunits likely does not require major rewiring of protein–protein interactions, unlike in the more curved smaller capsids.

A major question is why giant viruses evolved toward lower packaging densities. One possibility is that this happened due to the change of the genome delivery mechanism. With few exceptions, most giant viruses enter the host cell through endocytic or phagocytic pathways and, following the fusion between the viral and vesicular membranes, deliver the genome into the cytoplasm within a core particle (115). The energy stored in the form of genome condensation does not appear to play a role in this process, unlike in the case of viruses with smaller capsids, such as tectivirids (105). It is worth noting that unlike giant varidnavirians, large duplodnavirians (for example, jumbo phages) maintain high packaging densities irrespective of the capsid or genome size (107). In all likelihood, this trait is necessitated by the conservation of the genome delivery mechanism, which relies on the intracapsid pressure for DNA ejection into the cytoplasm in the case of prokaryotic duplodnavirians, and into the nucleus in the case of the animal herpesviruses (116). Thus, the switch from the direct DNA ejection into the host to the reliance on membrane fusion in most large varidnavirians apparently triggered the evolution toward lower packaging densities, which in turn enabled the evolution of expandable genomes.

The ecological and evolutionary consequences of the constraints imposed by the small capsids are such that viruses with nonexpandable genomes are forced to evolve mostly by optimizing existing components, following the *r*-like life strategies. By contrast, viruses with large, expandable genomes can both optimize existing functions and add new ones, leading to bursts of genetic innovation associated with the *K*-strategy. Accumulation of functionally diverse genes that support persistence, host manipulation, and ecological specialization enables alternation between the *r*-like and *K*-like strategies, depending on the ecological conditions.

## Conclusions

Giant viruses are a remarkable variety of MGE that encode a broad variety of functional systems some of which are considered to be hallmarks of cellular life forms, such as multiple components of the translation machinery. However, this striking complexity notwithstanding, giant viruses retain all the signatures of replicators in general and viruses in particular, that is, they are typical viruses, only with very large genomes and particles. The origin of giant viruses is not an enigma because their evolution from smaller, simpler viruses can be confidently traced in several lineages of realms *Varidnaviria* and *Duplodnaviria*.

The continuity in the genome and virion sizes between the “regular” and giant viruses highlights the lack of objective threshold of gigantism. Furthermore, even such arbitrary thresholds are not stable and with ever larger viruses being discovered, such as the putative megagenomoviruses (29), the bar to qualify as a giant virus would inevitably shift upward. The lack and, indeed, the impossibility of objective criteria to define gigantism prompts the question: Do giant viruses comprise a distinct class of biological entities different from other viruses? The objective answer is no. There is a continuum of viral genome and particle sizes, and all viruses, regardless of the size, share the same fundamental properties, rooted in their nature as replicators, that is, digital entities. Apart from the elusive and, arguably, unnecessary definition of viral gigantism, a major question is, what drove the evolution of extremely large genomes in some but not other, related groups of viruses. The choice between the *r* and *K* evolutionary strategies seems to be a conceptual answer, and the uncoupling of the genome size from the capsid size likely enabled adoption of the *K*-strategy, but reconstruction of concrete evolutionary scenarios requires deep investigation of the host biology and virus–host interactions. A major step in this direction would be unbiased assessment of the host ranges for varidnavirians across the genome size range. Culture-based approaches, although instrumental for elucidating intricate molecular details of virus–host interaction, are unlikely to achieve this task. Instead, it can be expected that culture-independent techniques, such as single-cell RNA sequencing (117), single-cell genomics (118), and metagenomic Hi-C (119), will provide major insights into giant viruses and their hosts, allowing more informed evaluation of factors driving viral evolution.

## Data Availability

There are no data underlying this work.
